# Affect, Behaviors of Children With Intellectual Disabilities and Parents' Coping Strategies During the COVID-19 Pandemic

**DOI:** 10.3389/fpsyt.2022.822908

**Published:** 2022-05-20

**Authors:** Minjie Ma, Xiao Wang, Peiyu Qi, Tingzhao Wang

**Affiliations:** ^1^Department of Special Education, School of Education, Shaanxi Normal University, Xi'an, China; ^2^Xi'an Mangya School, Xi'an, China; ^3^School of Education and Psychology, Hainan Normal University, Haikou, China

**Keywords:** COVID-19, affect, children with intellectual disabilities, coping strategies, problem behaviors

## Abstract

**Background:**

In early 2020, the COVID-19 pandemic emerged. To prevent the spread of the virus, China implemented restrictions on going out and ensured that people stayed at home. This study aims to investigate the affect and behaviors of children with intellectual disabilities (ID) during the lockdown. The informal coping strategies adopted by parents and their effects were further evaluated.

**Methods:**

In this study, a total of 457 parents of children (mean age: 14.82 years ± 1.96) with ID in 12 provincial administrative regions across China were surveyed online using the Positive and Negative Affect Scale and our own questionnaire on daily behaviors, problem behaviors and informal coping strategies.

**Results:**

During the COVID-19 pandemic, the positive affect (PA) score was significantly higher than that of negative affect (NA) (*p* < 0.001). Some children experienced mostly positive changes in sleep (16.63%), communication (14.66%), and diet (5.69%). However, more than one-third (39.39%) exhibited problem behaviors such as hyperactivity. A significant correlation was found between affect and behavior. The informal coping measures adopted by parents were generally effective among affect and the relationship with problem behaviors.

**Conclusions:**

The affect of the children with ID at home was mainly positive. The overall behaviors (diet, sleep, and communication) were good, but there were problem behaviors. Effective coping strategies are associated with higher PA, lower NA, and fewer problem behaviors. The greater the number of effective coping strategies, the lesser the problem behaviors.

## Introduction

In 2020, the COVID-19 pandemic broke out rapidly and spread all over the world. To effectively contain the pandemic, all Chinese provinces launched a Level I response to major public health emergencies and stopped all assembly and gathering activities. The Spring Festival holiday was extended from the January 28 to February 2, 2020 (1). People made efforts to fight against the pandemic by staying home both from work and social activities (2). According to clinical stress theory, long-term home isolation limits the physical activities of individuals, which causes adverse effects on their physical state, their affect, and their behavior (3–5).

The COVID-19 pandemic resulted in a rapid shutdown of social life, creating a closed environment that may have impacted both the mental and emotional health of teenagers with intellectual disabilities (ID) (6), who are quite susceptible to emotional and problem behaviors (7–9). Affected by physical and psychological conditions, children with ID are quite different from those without ID in the expression of subjective effects, such as generally poor emotional management ability, slower development of emotional ability (9), and even some defects (8). In the field of emotional research, positive affect (PA) and negative affect (NA) are important embodiments of mental health and psychological efficacy. Excessive NA, such as anxiety and depression, predicts higher social and behavioral problems (10); further, it is associated with avoidance coping strategies, positively correlated with social anxiety (11), and closely related to eating disorder tendency (12). Emotional problems or mood disorders in children may induce other mental disorders, cause behavioral disorders, and even increase the risk of suicide (13). Therefore, for children with ID who have more emotional problems, it is important to detect the onset of NA and find ways to improve PA and reduce NA to maintain their good emotional state.

During the COVID-19 pandemic, more than half of people with ID reported more mental health problems in Chile, and that number was about 41% in the United States (14). A recent study confirmed the severely negative psychological impact on psychiatric patients due to the strict lockdown measures (15). In a telephone interview of vocational school students with developmental disabilities, the majority of whom had mild ID, more than one-third reported mild or more severe symptoms of anxiety and depression, with girls being more affected (16). At the same time, more problem behaviors were also reported by parents of children with ID (17, 18). A group of parents stated in fact that their child's problem behaviors had been their biggest challenge since the stay-at-home order went into effect (18).

As a result of the pandemic, more than 75% of families of children with ID lost at least one therapy or educational service (18, 19). In cases where professionals could not provide timely support, the possibly unhealthy conditions of children with ID presented a substantial challenge for guardians (20, 21). A survey conducted during the pandemic showed that relative to carers of children without ID, carers of both children and adults with ID had significantly greater levels of defeat/entrapment, anxiety, and depression. The differences were two to three times greater than reported in pre-pandemic studies (22).

Despite the enormous difficulties, parents have taken steps to cope with the challenges. Parents from California and Oregon have implemented behavioral strategies, routines, engaging in enjoyable activities, or finding fun activities to do (18). It should be noted that coping strategies such as seeking outside help and professional support (23) are not readily available during the COVID-19 pandemic. However, emergency situations (such as challenging behaviors) in families of exceptional children need to be dealt with in time to prevent more serious problems. A meta-analysis of emotional and problem behaviors of children with autism showed that, when faced with extreme situations (e.g., outbursts, meltdowns), parents will comfort them with positive and gentle words, physical acts such as hugs, or remove them from the situation (24). This is consistent with the previous conclusion that positive parenting strategies (such as praise and positive non-verbal responses) can effectively improve children's compliance and decrease inappropriate behaviors (25, 26). O'Nions et al. (24) further pointed out that, when faced with problem behaviors, the diversionary methods used by parents are effective and can even prevent outbursts in challenging situations, while negative parenting strategies such as yelling and conveying negative affect are often ineffective (27). However, it remains to be seen how effective these coping strategies are for children with ID, especially during this particular time. After all, there have been requests for support and services from parents to address such challenges during the pandemic (28). Before providing these services, we also need to figure out what parents themselves can do at home to make support strategies more targeted. To address this, it is necessary to study the specific affect and behaviors of children with ID (29), the coping strategies their parents have taken, and the effects of these strategies.

This study investigated the affect and behavior of children with ID at home during the novel coronavirus outbreak, as well as the coping strategies (including behavior, language and diversion) and effects adopted by their parents, to provide a reference for psychological intervention during this singular period, as well as subsequent school teaching, rehabilitation treatment, and family management.

## Materials and Methods

### Participants and Settings

This study took place in March 2020 following approval by the Ethics Committee of Shaanxi Normal University. The participants were recruited through social networks in special education schools or rehabilitation institutions.

The participants were parents of exceptional children, selected based on the following criteria: (a) raised at least one child with ID, (b) children were between 12 and 18 years old, (c) children had IQ < 70, and (d) they voluntarily agreed to participate in this study.

Before the formal study, we conducted a pilot study with 16 parents and four teachers in two classes of children with ID. It helped to address any potential weaknesses of the survey items and included checking for clarity of wording, formatting, appropriateness of the number, labeling of response options, participants' acceptance of the questions, and so forth (30). This helped to improve the validity and reliability of the “Questionnaire on Daily Behaviors, Problem Behaviors of Children with ID and Parents' Informal Coping Strategies.”

We used items in the questionnaire to screen out participants such as, “Do you have children with ID in your family?” and “What is your child's level of ID?” Only those parents who met the criteria were included in the follow-up survey.

In this study, we screened out 457 exceptional children, including 262 boys (57.3%) and 195 girls (42.7%), from 12 provincial administrative regions of China. The average age of the participant children was 14.82 years (SD = 1.96, range = 12–18 years). The parents of these children completed the online questionnaire.

### Instruments

#### Positive and Negative Affect Scale

We used the Positive and Negative Affect Scale (PANAS) developed by Watson et al. (31) and revised by Huang et al. (32), with a 5-point Likert-type scale (1 = “almost none” to 5 = “extremely”). The scale is divided into two dimensions: positive affect (PA) and negative affect (NA). Each dimension consists of 10 questions. The results of confirmatory factor analysis showed that the model fit well (χ^2^/*df* = 2.40, *RMSEA* = 0.06, *TLI* = 0.92, *CFI* = 0.93), and the two-factor structure of the scale was confirmed. The average score of 10 items corresponding to each factor was taken. The Cronbach's alpha (α) coefficient was 0.87 for PA and 0.85 for NA.

#### Questionnaire on Daily Behaviors, Problem Behaviors of Children With ID and Parents' Informal Coping Strategies

We constructed a scale (Questionnaire on Daily Behaviors, Problem Behaviors of Children with ID and Parents' Informal Coping Strategies) to measure the behavior of children with ID and the effects of the informal coping strategies. The scale includes three aspects: daily behavior, problem behaviors, and informal coping strategy.

Daily behaviors and their changes were measured in three aspects: how were the children with ID eating at home during tough times; how were they sleeping during this time; how were they communicating with other family members. The first three questions examined the changes during the pandemic, and the participants rated each item on a 3-point scale (1 = better, 2 = no change, 3 = worse).

When we measured the specific problem behaviors, we used the five dimensions of the Aberrant Behavior Checklist (ABC) introduced by Ma et al. (33); additionally, the results of the pilot study with parents were used to identify more problem behaviors in the study. Finally, we indicated six main problems: hyperactivity, stereotyped behavior, inappropriate speech, violent behavior, sluggishness, and refusal to see people.

Informal coping strategies include behavioral comfort, language comfort, and diversion. *Behavioral comfort* comes from touch care, which refers to improving the emotional state and behavioral performance of teenagers with ID through touching, hugging, and other physical contacts (34, 35). *Language comfort* refers to positive comfort based on respect using a relaxed gentle tone (35). The specific ways to divert attention are based on the result of the pilot study with parents, which include watching TV, eating, playing on mobile phones, playing games, exercising, playing with toys, listening to music, writing homework, reading books, and other methods. The effects of the coping strategies are divided into two types, 1 = effective and 0 = ineffective, which were reported by the parents of children with ID.

### Data Analyses

Data were coded and analyzed using the IBM SPSS Statistics 23. Descriptive statistics were computed for each variable. The correlation analysis, independent sample *t*-test, and one-way analysis of variance (ANOVA) were used to test the relation of affect, behaviors, and the effect of coping strategies. Mplus 7.4 was used for confirmatory factor analysis of PANAS.

## Results

### Affect and Behaviors

During the pandemic, the PA scores of children with ID were significantly higher than those of NA (*t* = 17.71, *p* < 0.001). In addition, 16.63% of children with ID experienced changes in sleep, 14.66% experienced changes in communication, and 5.69% experienced changes in diet. These changes were mostly positive, such as communicating better and more frequently with families. Furthermore, 39.39% of the children with ID displayed problem behaviors at home. The top three problem behaviors were hyperactivity (19.91%), stereotyped behavior (12.04%), and inappropriate language (11.38%). “Hyperactivity and overactivity” has become one of the main problem behaviors at home of children with ID during the pandemic.

The results of ANOVA are shown in Table 1. Children with ID, whose sleep and diet worsened reported higher NA (*p* < 0.05). Better sleep and diet may reduce NA (*p* < 0.05). The problem behaviors were counted according to the number of occurrence categories (between 0 and 5). The number of problem behaviors was negatively correlated with PA (*r* = −0.11, *p* < 0.05) and positively correlated with NA (*r* = 0.36, *p* < 0.001). The more problem behaviors there were, the lower the PA, and the higher the NA.

**Table 1:** ANOVA for the differences of affect among changes of daily behaviors (*N* = 457).

	**Sleep**	**Communication**	**Diet**
Positive affect	*F* = 0.90	*F* = 2.37	*F* = 0.92
Negative affect	*F* = 4.12^*^	*F* = 0.04	*F* = 12.43^***^

* *p < 0.05*;

*** *p < 0.001*.

### Affect and Informal Coping Strategy

To mollify children with ID at home during the pandemic, parents have adopted informal coping strategies such as behavioral comfort (*N* = 362), language comfort (*N* = 399), and diversion (*N* = 444). In parents' opinion, the most effective strategy is diversion, followed by behavioral comfort and language comfort. Among the several attention-diverting strategies, watching TV (*N* = 209), eating (*N* = 135), and playing with mobile phones (*N* = 140) were the preferred ones. The most effective strategies to divert children's attention are listening to music and performing sports, though the above two were not the most preferred strategies by parents. Table 2 portrays the data of informal coping strategies.

**Table 2:** Informal coping strategies adopted by the parents of children with intellectual disabilities.

**Coping strategies**	**Behavioral comfort**	**Language comfort**	**Diversion (*****N*** **=** **444, Effective rate** **=** **67.63%)**								
			**Watching TV**	**Playing with mobile phones**	**Eating**	**Performing sports**	**Listening to music**	**Playing games**	**Doing homework**	**Playing with toys**	**Reading**
Samples	362	399	209	140	135	134	115	92	86	71	50
Effective rate	61.60%	57.14%	65.07%	70.71%	61.94%	75.37%	79.13%	69.57%	56.98%	66.20%	58.00%

The results of independent sample *t-test* are shown in Table 3. The PA and NA scores of different coping effects were significantly different. The PA of effective coping strategies was significantly higher than that of ineffective coping strategies, while the NA was significantly lower in ineffective coping strategies.

**Table 3:** *T*-test of the effect of informal coping strategies and affect in children with intellectual disabilities.

	**Behavioral comfort**	**Language comfort**	**Diversion of attention**
	**(*N* = 362)**	**(*N* = 399)**	**(*N* = 444)**
Positive affect	*t* = 4.30^***^	*t* = 4.65^***^	*t* = 3.94^***^
Negative affect	*t* = −4.03^***^	*t* = −6.16^***^	*t* = −2.08^*^
Problem behaviors	*t* = −4.45^***^	*t* = −5.40^***^	*t* = −2.90^**^

* *p < 0.05*;

** *p < 0.01*;

*** *p < 0.001*.

### Problem Behavior and Informal Coping Strategy

Children with ID performed well at home during the pandemic and had few negative changes in sleep, diet, and communication. However, the incidence of problem behaviors was high. Therefore, we investigated the specific effects of three types of informal coping strategies. The results are shown in Table 3.

Our results indicate that the three types of coping strategies had inhibitory effects on problem behaviors. Children with ID whose coping strategies were effective reported fewer problem behaviors. When problem behaviors occurred, the coping effectiveness of language comfort, behavioral comfort, and diversion decreased sequentially (*|t|*: 5.40> 4.45 > 2.9).

Furthermore, the number of effective informal coping strategies adopted by each family was calculated (between 0 and 3). The correlation between the number of effective coping strategies and the number of problem behaviors was then calculated. A significant correlation was found between them, *r* = −0.27, *p* < 0.001. The greater the number of effective coping strategies used by children with ID, the fewer the problem behaviors reported.

## Discussion

Major public health emergencies can profoundly affect people's psychology negatively. Thus, it should come as no surprise that the pandemic has led to many psychological and neuropsychiatric problems for both ordinary people and patients (3, 36–39). However, the results of this study show that the emotional state of children with ID was not significantly worse off, and that children with ID generally exhibited good behavior, although this result may be due to the fact that the survey was conducted just after the Spring Festival. The Spring Festival is the grandest festival of the Chinese year, similar to the Christmas and New Year celebrations in Western countries. The festival offers opportunities to eat a lot of delicious food during the Reunion Dinner, while the custom of family reunion during the Spring Festival combined with home isolation during the pandemic might create more opportunities for family members to get along and communicate with each other. In the happy atmosphere of the Spring Festival, even though the children with ID were restricted from going out, they had significantly more PA than NA; sleep, communication, and diet also showed positive changes.

The overall good behavioral performance of children with ID was mutually correlated with better affect. Therefore, paying attention to the emotional changes of children with ID can predict possible problem behaviors and assist in carrying out suitable intervention (40). Similarly, for children with ID, maintaining a stable emotional and psychological state by developing regular living habits and reducing interference from negative information may help in both their education and social interaction (41). An effective coping strategy adopted by the parent can also affect emotions. In the process of long-term medical consultation and rehabilitation treatment, the parents have a certain understanding of the children's condition and have gradually found simple and effective informal coping strategies suitable for their children. Just as the parents noted, ‘I was able to take care of my child very well, I knew what he/she wanted and how to deal with it, if he/she was too excited to show problem behaviors, I would hug him/her in my arms and sing a song at his/her ear, and he/she would calm down. These skills may contribute to their ability to take good care of children with ID when staying at home during the pandemic. Therefore, parents should also take time to pay attention and for informal coping strategies during non-epidemic periods, as such attention may help young people with ID establish and maintain a positive emotional state.

Some of the problem behaviors of children with ID during the pandemic may have been caused by the combined effects of physical and psychological barriers as well as the pandemic itself. One in five parents reported problem behaviors of hyperactivity, which perhaps are affected by restrictions on going out. Children are generally active outside, so now that outside activities are moved indoors (such as running and jumping), it might give parents the impression that the child is hyperactive and overactive. However, maybe the self-hindrance (such as behavior abnormality that may result from ID) is the major factor in problem behaviors. For example, children with ID and autism are prone to problem behaviors such as aggression and self-mutilation (42). Staying at home during the pandemic may have an adverse impact on these problem behaviors but may not be the essential cause. There are also people with ID who take antipsychotic drugs daily to suppress problem behaviors (43, 44). Affected by the pandemic, many were not able to return to the clinic on time to maintain their drug routine, leading to problems such as insufficient drug reserves and irregular medicinal uptake schedules (20), which may aggravate old problem behaviors or induce new ones. Alternatively, if parents do not have the right coping strategies and external support, they may not be able to cope with the problem behaviors of children with ID. The lack of formal services and parent-mediated training may lead to problem behaviors for children with ID (18).

Most parents adopted at least one coping strategy. The self-reported data on affect and problem behaviors showed that coping strategies were effective. However, there remains debate about which strategy is more effective. Parents self-reported the order of effectiveness of coping strategies as diversion, behavioral comfort, and language comfort. However, when examining the relationship between the effectiveness of coping strategies and problem behaviors, it is exactly the opposite of the previous order. The problem behaviors corresponding to the effectiveness of diversion, behavioral comfort, and language comfort were gradually reduced. This may be because the children with ID who develop problem behaviors are more difficult to raise than those who do not. Informal coping strategies adopted by parents in the face of parenting dilemmas during lockdown are more likely to be effective when there are no problem behaviors. In addition, what parents self-reported as effective might not necessarily have been effective. In the absence of formal services and parental management during the lockdown, the effectiveness of informal coping strategies has not been verified by professionals. For children with ID, the effectiveness of a single coping strategy is uncertain (45). However, adopting several coping strategies at the same time can possibly lead to better intervention outcomes (46). When managing outbursts, the parents can try to comfort the child by telling them: “It's OK” (language comfort), hug them gently (behavioral comfort), and use the specific activities or “must-have” items to distract them (diversion) (24).

### Limitations and Future Research

For children with ID, their parents play a more pivotal role in their growth at such an unusual time. However, this study only investigated some of the simplest informal coping strategies in the parenting process. In other words, future research should use experimental design rather than just observational data to verify the effectiveness of coping strategies as well as their long-term effects. For example, researchers can use panel research to track which informal coping strategy is more effective. They could also conduct a mixed study to make the conceptualization of informal coping strategies clearer and more accurate. IQ score is a key factor for children dealing with their affairs, so how to establish special family coping strategies for children with different intelligence levels may be another meaningful research direction. Simultaneously, the function of schools and social institutions (e.g., community and rehabilitation facilities) should also be considered. They should live up to their responsibilities and provide professional support to both parents and children to the best of their abilities. This will bring significant direct and indirect benefits to the children.

## Conclusion

During the early pandemic, children with ID at home exhibited good affect and daily behaviors. However, 39.39% of the participants still had problem behaviors such as hyperactivity. We found that the greater the number of effective informal coping strategies, the lesser the problem behaviors exhibited. Future research should focus on effective coping strategies, and use this as a basis for parent management and training.

## Data Availability Statement

The raw data supporting the conclusions of this article will be made available by the authors, without undue reservation.

## Ethics Statement

The studies involving human participants were reviewed and approved by Shaanxi Normal University. The patients/participants provided their written informed consent to participate in this study.

## Author Contributions

MM: conception, design of the work, data acquisition, curation and analysis, writing—original draft, and writing—reviewing and editing. XW: translation and writing—reviewing and editing. PQ and TW: supervision and writing—reviewing and editing. All authors read and approved the final manuscript.

## Funding

This work was supported by the National Social Science Fund of China (Grant Number: 17XTY007), the Humanities and Social Sciences Fund of the Ministry of Education in China (Grant Number: 20YJA890019), and the Fundamental Research Funds For the Central Universities in China (Grant Number: 2019TS044).

## Conflict of Interest

The authors declare that the research was conducted in the absence of any commercial or financial relationships that could be construed as a potential conflict of interest.

## Publisher's Note

All claims expressed in this article are solely those of the authors and do not necessarily represent those of their affiliated organizations, or those of the publisher, the editors and the reviewers. Any product that may be evaluated in this article, or claim that may be made by its manufacturer, is not guaranteed or endorsed by the publisher.
